# Supplementary material for: Preparation and Properties of Hydrophobic Polyurethane Based on Silane Modification
Source: Polymers (Basel). 2023 Mar 31;15(7):1759. doi: 10.3390/polym15071759 (PMC10096977; doi:10.3390/polym15071759)
Supplement: Supplementary file 1 [file polymers-15-01759-s001.zip › polymers-2297963-supplementary.docx]

# Supplementary Materials

**Table S1.** Particle size, polydispersity index (PDI), Zeta potential and stability of SWPUA emulsion with different silicon content.

| **Sample** | **Silicon content/%** | **Particle size/nm** | **PDI/%** | **Zeta potential/mV** | **Centrifugal stability** | **Storage stability/moon** |
| --- | --- | --- | --- | --- | --- | --- |
| SWPUA-0 | / | 68.35 | 22.3 | 55.27 | No settling | >6 |
| SWPUA-5 | 5.0 | 101.93 | 25.8 | 54.47 | No settling | >6 |
| SWPUA-10 | 10.0 | 76.29 | 23.5 | 53.23 | No settling | >6 |
| SWPUA-15 | 15.0 | 103.31 | 24.0 | 52.30 | sedimentation | <6 |
| SWPUA-20 | 20.0 | 107.83 | 26.6 | 50.33 | sedimentation | <6 |

**Table S2.** The serial numbers in the radar diagram and their corresponding references.

| **No.** | **1** | **2** | **3** | **4** | **5** |
| --- | --- | --- | --- | --- | --- |
| Ref | [42] | [30] | [43] | [18] | [20] |
| **No.** | **6** | **7** | **8** | **9** | **10** |
| Ref | [44] | [12] | [45] | [46] | [47] |
| **No.** | **11** | **12** | **13** | **14** | **15** |
| Ref | [48] | [49] | [50] | [25] | [51] |
| **No.** | **16** | **17** | **18** | **19** | **20** |
| Ref | [52] | [53] | [54] | [55] | [31] |
